# Protein‐like Enwrapped Perylene Bisimide Chromophore as a Bright Microcrystalline Emitter Material

**DOI:** 10.1002/anie.201907618

**Published:** 2019-08-12

**Authors:** David Schmidt, Matthias Stolte, Jasmin Süß, Andreas Liess, Vladimir Stepanenko, Frank Würthner

**Affiliations:** ^1^ Institut für Organische Chemie & Center for Nanosystems Chemistry Universität Würzburg Am Hubland 97074 Würzburg Germany

**Keywords:** crystal engineering, dyes, fluorescence quantum yield, perylene bisimides, solid-state emitters

## Abstract

Strongly emissive solid‐state materials are mandatory components for many emerging optoelectronic technologies, but fluorescence is often quenched in the solid state owing to strong intermolecular interactions. The design of new organic pigments, which retain their optical properties despite their high tendency to crystallize, could overcome such limitations. Herein, we show a new material with monomer‐like absorption and emission profiles as well as fluorescence quantum yields over 90 % in its crystalline solid state. The material was synthesized by attaching two bulky tris(4‐tert‐butylphenyl)phenoxy substituents at the perylene bisimide bay positions. These substituents direct a packing arrangement with full enwrapping of the chromophore and unidirectional chromophore alignment within the crystal lattice to afford optical properties that resemble those of their natural pigment counterparts, in which chromophores are rigidly embedded in protein environments.

Solid‐state fluorescent organic materials^1^, ^2^, ^3^ are of considerable interest as they enable a broad variety of applications in (opto‐)electronics, for example, organic light emitting diodes (OLEDs),^4^ waveguiding,^5^, ^6^ solid‐state lasers^7^, ^8^, ^9^ luminescent sensors,^10^ or as fluorescent labels for (bio)medical research^11^ as well as security printing technologies.^12^ Unfortunately, while pigments are the preferred colorants for these applications compared to dyes owing to their superior thermal, photochemical, and chemical robustness, only very few pigments exhibit a decent fluorescence.^13^ In contrast to the bright luminescence with quantum yields (*Φ*_F_) of up to unity for many organic chromophores in diluted solutions, intermolecular interactions in the solid state open up a multitude of non‐radiative relaxation pathways that quench the fluorescence emission.^14^, ^15^, ^16^

Owing to their high molar absorptivity and excellent chemical and thermal stability as well as lightfastness, perylene bisimides (PBIs) are an exceptional class of organic colorants, both as soluble dyes with fluorescence quantum yields up to unity and as pigments.^17^ However, all commercial PBI pigments with their red, maroon, and black shades are non‐fluorescent,^18^ and it remains a challenge to overcome the prevailing fluorescence quenching pathways and to design PBI‐based solid‐state emitter materials that maintain the intense fluorescence that is commonly observed for PBI dyes in diluted solutions. Systematic studies on PBI dye aggregates carried out in our laboratory as well as others during the last decade have identified excimer formation,^19^, ^20^ symmetry‐breaking charge separation,^21^ and several triplet state population processes^22^ as the main fluorescence‐quenching pathways. Because all of these processes are favored by close π–π‐stacking of PBI dyes,^23^ much effort has been dedicated to the steric shielding of the chromophore core at imide, headland, or bay positions^24^, ^25^, ^26^, ^27^, ^28^ as well as by isolating individual chromophores in a host matrix.^29^ Other recent approaches include the organization of perylene dyes in orthogonally crossed arrangements or at magic angle slipping in which the excitonic coupling vanishes.^30^ Interestingly, while by these measures solid‐state fluorescence quantum yields of up to 59 % could indeed be achieved, the absorption and fluorescence spectra of the solid‐state materials in all examples still reveal substantial electronic interactions between the PBI dyes whose large π‐scaffolds appear to attract each other in sometimes rather unexpected ways with the concomitant deterioration of the fluorescence properties.

In this contribution, we report a sterically fully enwrapped.^31^, ^32^, ^33^, ^34^, ^35^ PBI derivative whose solid‐state absorption and emission properties fully match those in solution. Accordingly, monomer‐like vibronic progressions in the absorption and emission spectra and, most importantly, a fluorescence quantum yield close to unity under ambient conditions were obtained. This exciting result is rationalized by single crystal X‐ray analysis, which unveils a complete enwrapping of the PBI fluorophore in the solid state (Figure 1). Thus, a specific environment of the dye is created, quite similar to the embedding of chromophores in protein matrices of green fluorescent proteins^36^ or natural light‐harvesting pigment^37^ in which non‐fluorescent relaxation pathways are slowed down by chromophore isolation and rigidification, and high chromophore concentrations are realized without compromising the dyes’ desirable photo‐functional properties.

**Figure 1:**
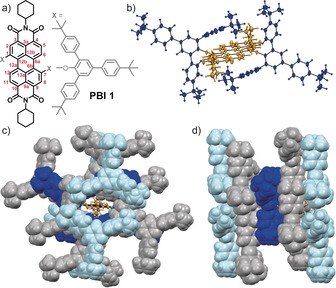
a) Chemical structure of **PBI 1** with labeling of the carbon atoms of the chromophore core (red) and b) its solid‐state molecular structure determined by single crystal structure analysis (ellipsoids set to 50 % probability).^50^ Packing arrangement of **PBI 1** in the solid state as viewed along the c) long and d) short molecular chromophore axis (ellipsoids of the PBI π‐scaffold set to 50 % probability and colored in orange and its 2,4,6‐tris(4‐*tert*‐butylphenyl)phenoxy substituents colored in dark blue; 2,4,6‐tris(4‐*tert*‐butylphenyl)phenoxy substituents of adjacent chromophores are illustrated as space filling model and are alternatingly colored in gray and light blue).

To synthesize a solid‐state emitting material with fully enwrapped PBI fluorophores, we introduced bulky 2,4,6‐tris(4‐*tert*‐butylphenyl)phenoxy substituents at the 1,7‐bay positions of regioisomerically pure *N*,*N*′‐dicyclohexyl‐1,7‐dibromoperylene‐3,4:9,10‐tetracarboxylic acid bisimide (Figure 1 a). By applying typical reaction conditions for nucleophilic aromatic substitution reactions, **PBI 1** could be isolated as a bright fluorescent and thermally highly stable (up to >350 °C) solid‐state material in 78 % yield after purification by column chromatography (Supporting Information, Scheme S1). The new solid‐state emissive PBI derivative was unambiguously characterized by ^1^H and ^13^C NMR spectroscopy, high‐resolution mass spectrometry, cyclic voltammetry, and elemental analysis (Supporting Information, Figure S1). Furthermore, solvent‐free single crystals suitable for X‐ray diffraction (XRD) analysis could be grown by slow evaporation of a dichloromethane/hexane 3:7 solution of **PBI 1** at room temperature, providing unique insights into its molecular structure and packing arrangement in the solid state (Figures 1 b–d and Supporting Information, Table S1 and Figure S2).

Similar to other 1,7‐diphenoxy substituted PBI derivatives,^27^, ^28^ **PBI 1** is characterized by an almost planar π‐scaffold with dihedral angles of 1.8(4)° and 0.5(3)° for the four bay (∡(C1‐C12b‐C12a‐C12)) and four inner carbon atoms (∡(C13a‐C12a‐C12b‐C13b); for numbering see Figure 1 a). Owing to the crystallographic inversion center located in the center of gravity of the molecule (space group $P\bar{1}$ ), the twist angle between both naphthalene mono‐imide subunits is 0°. The phenoxy substituents are bent towards opposing faces of the PBI π‐scaffold with dihedral angles of 31.1(3)° (∡(C2‐C1‐O‐C_aryl_) and ∡(C8‐C7‐O‐C_aryl_)), thereby efficiently shielding the chromophore's π‐scaffold (Figure 1 b). Owing to the bulky 4‐*tert*‐butylphenyl moieties, the individual chromophores are well‐separated in the solid state with an interplanar distance of 8.1 Å and a transversal and longitudinal displacement of 3.7 Å and 7.9 Å, respectively (Supporting Information, Figure S2). The packing arrangement of **PBI 1** is characterized by strictly parallel oriented molecules with several C−H⋅⋅⋅O and C−C⋅⋅⋅π interactions and a total void volume of 34 Å^3^. Most importantly, in addition to the jacketing provided by each two *tert*‐butylphenyl units attached at the *ortho*‐position of the 1,7‐bay‐substituents (dark blue colored, Figures 1 b–d), full chromophore enclosure is realized by *tert*‐butylphenyl units of the neighboring chromophores (grey and light blue colored, Figures 1 c,d). This desired feature became possible by the molecular design with three *ter*t‐butylphenyl units attached to the 2,4,6‐positions of the chromophore‐bound phenoxy units. The perfect chromophore embedment is corroborated by the absence of co‐crystallized solvent molecules and an appreciably high density for an organic crystal of 1.156 g cm^−3^. All these structural features can likewise be found in the isolated bulk material according to powder XRD analysis (Supporting Information, Figure S3 d) and do account for the bright solid‐state luminescence of **PBI 1** (see below).

The optical properties of **PBI 1** were investigated by UV/Vis absorption and fluorescence spectroscopy, both in dichloromethane solutions and in the solid state (Figure 2 and Supporting Information, Figures S3 a–c). The solid‐state absorption profile of **PBI 1** in reflection mode was determined on an ensemble of microcrystals in a BaSO_4_ trituration, applying the Kubelka–Munk theory (Figure 2).^38^ Both absorption spectra reveal sharp S_0_→S_1_ transitions located at 559 nm (solution) and 557 nm (solid), respectively, each with a well‐resolved vibronic progression at intervals of 1370 cm^−1^ and a molar extinction coefficient (*ϵ*_max_) of 7.2×10^4^ m ^−1^ cm^−1^ for the lowest energy 0,0‐vibronic transition (Table 1). The monomer transition is bathochromically shifted by 1200 cm^−1^ (35 nm, solution) compared to the bay‐unsubstituted *N*,*N*′‐dicyclohexyl‐3,4:9,10‐perylenetetracarboxylic acid bisimide owing to the electron‐donating character of the phenoxy substituents.^28^ The higher energetic S_0_→S_2_ transitions can be observed at 410 nm (solution) and 408 nm (solid), respectively, which is absent in the absorption spectrum of the parent chromophore. Thus, owing to the absence of significant inter‐chromophore interactions in the solid state, the absorption spectral features are essentially identical to those in solution with the exception of a slightly reduced ratio for the intensities of the apparent 0‐0 and 0–1 vibronic transitions (A_0‐0_/A_0‐1_) of 1.61 (solid) compared to 1.79 (solution), which may suggest a very weak exciton coupling.^39^

**Figure 2:**
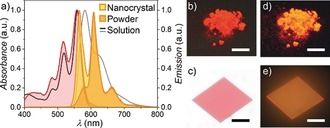
a) Absorption (red) and emission (orange, yellow) of **PBI 1** as crystalline powder as well as an individual microcrystal at room temperature and absorption (black solid line) and fluorescence (gray solid line) spectra of **PBI 1** in dichloromethane solution (*c*_0_=10^−7^ m) at 298 K. Photographs of crystalline powder (b,d; scale bar=1 cm) on a black surface and an individual microcrystal (c,e; scale bar=50 μm) on quartz of **PBI 1** under ambient light (b,c) and upon UV irradiation (d,e) that were used for spectroscopic investigations.

**Table 1:** Spectroscopic properties of **PBI 1** in dichloromethane solution and in the crystalline solid state at 298 K.

	*λ*_abs_	*FWHM*_abs_^[a]^	*λ*_em_	*FWHM*_em_^[a]^	Δ*ṽ*_Stokes_	*Φ*_F_	*τ*_F_
	[nm]	[cm^−1^]	[nm]	[cm^−1^]	[cm^−1^]	[%]	[ns]
CH_2_Cl_2_	559	750	581	1130	680	100	5.20
Solid	557^[b]^	1000	565^[c]^	570	260	>90^[b]^	7.70^[b]^

[a] *FWHM* was derived as twice the distance between the maximum to the closest edge at half‐maximum of the unsymmetrically shaped absorption or emission band to prevent falsification by overlapping transitions. [b] Ensemble property of crystalline material. [c] Single microcrystal investigated with an optical polarization microscope equipped with a fiber‐coupled CCD spectrometer.

For an accurate comparison of the fluorescence properties of **PBI 1** in solution and in the solid state, the intrinsic steady‐state emission spectra have to be acquired without any contamination by reabsorption, which is rather challenging for solid‐state samples of chromophores with high tinctorial strengths (*ϵ*_max_) and small Stokes shifts (Δ*ṽ*_Stokes_). Whereas highly diluted solutions of **PBI 1** (*OD*<0.05; *c*_0_<10^−6^ m) are routinely investigated using standard fluorescence spectrometers, the spectroscopic investigations on micrometer‐sized single crystals of **PBI 1** were performed with an optical polarization microscope equipped with a fiber optic‐coupled CCD spectrometer. Thus, single crystals of different sizes and thicknesses were grown from dichloromethane/hexane 3:7 solutions and subsequently investigated (Supporting Information, Figure S4) to obtain nearly reabsorption‐free solid‐state emission spectra (Figure 2). These data are most accurate and allow for a quantitative analysis of the intrinsic solid‐state fluorescence properties of **PBI 1**.

In contrast to previously reported 1,7‐diphenoxy‐substituted PBI derivatives,^27^, ^28^ **PBI 1** displays a solid‐state fluorescence spectrum that is an almost perfect mirror image of its absorption profile with distinct vibronic progressions at 565, 608, and 663 nm (each separated by 1360 cm^−1^). It is remarkable that even the emission spectrum of **PBI 1** in dichloromethane solution is more broadened, less structured, and more red‐shifted by 490 cm^−1^ (Figure 2 and Supporting Information, Figure S3 a), which hitherto could only be observed for natural pigments (i.e., protein‐encapsulated chromophores)^30^, ^31^ but not for synthetic pigments. Likewise, the Stokes shift (Δ*ṽ*_Stokes_=260 cm^−1^) and the full width at half maximum (*FWHM*_em_=570 cm^−1^) of the shortest wavelength emission of microcrystalline **PBI 1** is approximately half of that of its monomer emission in solution (Table 1). These observations can be rationalized by a rigidification of the chromophore π‐scaffold by its enclosing environment in the solid matrix, thereby preventing structural relaxations of the excited molecules (as well as their environment) as it is given in solution. In accordance with this interpretation, an increased fluorescence lifetime of 7.70 ns was observed for the crystalline sample compared to the value of 5.20 ns in dichloromethane solution (Supporting Information, Figure S3 b). Most importantly, the bright fluorescence of **PBI 1** in solution (*Φ*_F_=100 %) is also retained in the solid state with a fluorescence quantum yield *Φ*_F_ of at least 90 % after correcting the apparent solid‐state emission quantum yield (*Φ*_F_*=82 %) for unavoidable reabsorption losses (Figure 2 c and Supporting Information, Figure S3 c).^40^

To gain further insights into the optical properties of this unique highly luminescent solid‐state material, oriented microplatelets of **PBI 1** were grown on quartz substrates^41^ from dichloromethane/hexane 4:6 solutions and their anisotropy was investigated by optical polarization (fluorescence) microscopy (Figure 3 and Supporting Information, Figures S5–6). The crystallinity of these samples was demonstrated by selected area electron diffraction (SAED) and out‐of‐plane XRD experiments performed on microcrystals grown on a carbon‐coated copper grid, confirming an identical solid‐state packing arrangement as determined by single crystal structure analysis (see above; Supporting Information, Figures S7 a–c). Independent on the substrate, the rhomboid crystals exhibit edge lengths of several tens of micrometers and angles of 107° and 73° (Figure 3 b), respectively, with thicknesses of several hundred nanometers according to AFM cross‐section analysis (Supporting Information, Figure S7 d–f). Accordingly, owing to the high tinctorial strength, the emission spectra of those microplatelets are partially contaminated by reabsorption (see above) and no absorption spectra could be recorded in transmission mode. SAED and XRD experiments unambiguously confirmed that the investigated microcrystals are oriented with their (010) plane parallel to the surface (Figure 3 b and Supporting Information, Figures S7 a–c). The fast‐growing directions of the crystals, which give rise to their rhomboidal shape, are presumably the (100) and (001) lattice planes as evidenced by the simulated Bravais–Friedel–Donnay–Harker (BFDH) morphology (Figure 3 b). The long molecular axes of the PBI chromophores and thus their S_0_→S_1_ transition dipole moments are unidirectionally aligned along the short rhomboid diagonal that intersects the long one at an angle of 86° (Figure 3 b). Accordingly, rhomboid crystals of **PBI 1** appear deep red in optical polarization microscopy when the transmitting light is polarized along the short rhomboid diagonal but become fully transparent when the polarization is rotated by 90° (Figure 3 b and Supporting Information, Figure S6). This behavior becomes even more obvious in polarization‐dependent fluorescence microscopy experiments starting with polarizer, analyzer, and short rhomboid diagonal all aligned parallel (Figures 3 a,c). By rotating the short diagonal out of this arrangement the bright orange fluorescence gradually decreases and is completely absent at an angle of 90° (Figures 3 a,c). Apparently, this process is characterized by a huge optical anisotropy as the emission intensity at 606 nm is reduced by 99 % at a perpendicular orientation (Figure 3 a). The high quality of the microcrystals and the perfect unidirectional alignment of the chromophores is thereby reflected in an outstanding dichroic ratio, *D*_606nm_, of 82 and an order parameter, *S*_606nm_, of 0.96.^42^, ^43^, ^44^

**Figure 3:**
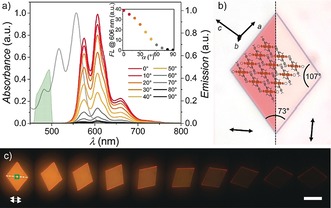
a) Polarization‐dependent fluorescence spectra of a **PBI 1** microcrystal on quartz displayed in (c). The gray solid line marks the absorption and the green area the excitation profiles of a band‐gap filter cube, respectively. Inset: Respective change of the emission strength at 606 nm. b) Optical microscopy images of a microcrystal for different polarizations (arrow=15 μm) of the transmitted light along (left) and perpendicular (right) to the long molecular axis including a schematic molecular arrangement. c) Fluorescence microcopy images of a microcrystal upon rotation with respect to parallel aligned polarizer (excitation) and analyzer (detection). The green square marks the area where the spectra were recorded. Scale bar=50 μm.

The spectroscopic data accordingly fully meet our expectations based on the packing features. Different from conventional pigments or other π‐conjugated thin‐film and solid‐state materials in which electronic couplings from closely packed chromophore units lead to alterations of the spectral features with the commonly observed fluorescence quenching, the outstanding optical properties of **PBI 1** are fully conserved and even improved in the solid state. Such features were hitherto exclusive for natural pigments in which evolutionarily optimized protein environments afford chromophore isolation and chromophore rigidification. For this reason, natural light‐harvesting pigments can have quite high dye densities to afford the desired high absorption cross sections for the capture of sunlight without undesired “concentration”‐quenching of the photo‐excited state. For **PBI 1**‐based thin films or crystals, the chromophore concentration is even higher than for any natural pigment because the chromophore part, that is, the perylene bisimide dye, constitutes 27.4 % of the total molecular mass of **PBI 1** (*c*=0.76 mol L^−1^;^29^ Supporting Information, Figure S9 a). For most protein‐based colorants this value is considerably smaller (Supporting Information, Figures S8 and S9), for example, 0.8 % for the green fluorescent protein,^45^ 0.54–0.85 % for rhodopsin,^46^, ^47^ 9.7 % for the Fenna–Matthews–Olson photosynthetic light‐harvesting protein complex.^48^, ^49^

In summary, we reported a unique perylene bisimide solid‐state emitter, which readily crystallizes from dichloromethane/hexane solutions to form rhomboid microcrystals with absolute solid‐state fluorescence quantum yields greater than 90 %. The sterically demanding threefold *tert*‐butylphenyl‐functionalized 1,7‐phenoxy substituents efficiently shield the planar chromophore core within the crystal lattice, providing complete wrapping and preventing electronic coupling between adjacent molecules, while maintaining an exceptional high chromophore concentration of more than 27 % in the solid state. This well‐defined intrinsic dilution of the chromophore core, like it is exclusively illustrated by many natural light‐harvesting pigments, accounts for its remarkable solid‐state luminescence and gives rise to a unique emission spectrum with a well‐resolved vibronic progression. The perfect unidirectional alignment of the chromophores within the single crystalline material enables a huge optical anisotropy with a dichroic ratio of 82, making **PBI 1** a highly attractive solid‐state material for photonic applications.

## Conflict of interest

The authors declare no conflict of interest.

## Supporting information

As a service to our authors and readers, this journal provides supporting information supplied by the authors. Such materials are peer reviewed and may be re‐organized for online delivery, but are not copy‐edited or typeset. Technical support issues arising from supporting information (other than missing files) should be addressed to the authors.

Supplementary

## References

[1] A. Kaeser , A. P. H. J. Schenning , Adv. Mater. 2010, 22, 2985–2997. 20535737 10.1002/adma.201000427

[2] B.-K. An , J. Gierschner , S. Y. Park , Acc. Chem. Res. 2012, 45, 544–554. 22085759 10.1021/ar2001952

[3] M. Shimizu , T. Hiyama , Chem. Asian J. 2010, 5, 1516–1531. 20583038 10.1002/asia.200900727

[4] M. C. Gather , A. Köhnen , K. Meerholz , Adv. Mater. 2011, 23, 233–248. 20976676 10.1002/adma.201002636

[5] Z. Yu , Y. Wu , Q. Liao , H. Zhang , S. Bai , H. Li , Z. Xu , C. Sun , X. Wang , J. Yao , H. Fu , J. Am. Chem. Soc. 2015, 137, 15105–15111. 26580969 10.1021/jacs.5b10353

[6] N. Chandrasekhar , R. Chandrasekar , Angew. Chem. Int. Ed. 2012, 51, 3556–3561; 10.1002/anie.201106652 22253053

[7] X. Wang , Q. Liao , Q. Kong , Y. Zhang , Z. Xu , X. Lu , H. Fu , Angew. Chem. Int. Ed. 2014, 53, 5863–5867; 10.1002/anie.201310659 24764282

[8] J. Gierschner , S. Varghese , S. Y. Park , Adv. Opt. Mater. 2016, 4, 348–364.

[9] M. G. Ramírez , S. Pla , P. G. Boj , J. M. Villalvilla , J. A. Quinta , M. A. Díaz-García , F. Fernández-Lázaro , A. Sastre-Santos , Adv. Opt. Mater. 2013, 1, 933–938.

[10] A. P. de Silva , H. Q. N. Gunaratne , T. Gunnlaugsson , A. J. M. Huxley , C. P. McCoy , J. T. Rademacher , T. E. Rice , Chem. Rev. 1997, 97, 1515–1566. 11851458 10.1021/cr960386p

[11] G. P. C. Drummen , Molecules 2012, 17, 14067–14090. 23192185 10.3390/molecules171214067 PMC6268024

[12] X. Hou , C. Ke , C. J. Bruns , P. R. McGonigal , R. B. Pettman , J. F. Stoddart , Nat. Commun. 2015, 6, 6884. 25901677 10.1038/ncomms7884 PMC4423226

[13] A. Dreuw , J. Plötner , L. Lorenz , J. Wachtveitl , J. E. Djanhan , J. Brüning , T. Metz , M. Bolte , M. U. Schmidt , Angew. Chem. Int. Ed. 2005, 44, 7783–7786; 10.1002/anie.200501781 16315162

[14] T. M. Figueira-Duarte , K. Müllen , Chem. Rev. 2011, 111, 7260–7314. 21740071 10.1021/cr100428a

[15] R. Katoh , K. Suzuki , A. Furube , M. Kotani , K. Tokumaru , J. Phys. Chem. C 2009, 113, 2961–2965.

[16] J. Shi , L. E. Aguilar Suarez , S.-J. Yoon , S. Varghese , C. Serpa , S. Y. Park , L. Lüer , D. Roca-Sanjuán , B. Milián-Medina , J. Gierschner , J. Phys. Chem. C 2017, 121, 23166–23183.

[17] T. Weil , T. Vosch , J. Hofkens , K. Peneva , K. Müllen , Angew. Chem. Int. Ed. 2010, 49, 9068–9093; 10.1002/anie.200902532 20973116

[18] K. Hunger , W. Herbst , Industrial Organic Pigments: Production, Properties, Applications , 3rd ed., Wiley-VCH, Weinheim, 2004.

[19] R. F. Fink , J. Seibt , V. Engel , M. Renz , M. Kaupp , S. Lochbrunner , H.-M. Zhao , J. Pfister , F. Würthner , B. Engels , J. Am. Chem. Soc. 2008, 130, 12858–12859. 18767851 10.1021/ja804331b

[20] J. Sung , P. Kim , B. Fimmel , F. Würthner , D. Kim , Nat. Commun. 2015, 6, 8646. 26492820 10.1038/ncomms9646 PMC4639892

[21] P. Spenst , R. M. Young , M. R. Wasielewski , F. Würthner , Chem. Sci. 2016, 7, 5428–5434. 30034681 10.1039/c6sc01574c PMC6021749

[22] S. W. Eaton , L. E. Shoer , S. D. Karlen , S. M. Dyar , E. A. Margulies , B. S. Veldkamp , C. Ramanan , D. A. Hartzler , S. Savikhin , T. J. Marks , M. R. Wasielewski , J. Am. Chem. Soc. 2013, 135, 14701–14712. 24011336 10.1021/ja4053174

[23] F. Würthner , C. R. Saha-Möller , B. Fimmel , S. Ogi , P. Leowanawat , D. Schmidt , Chem. Rev. 2016, 116, 962–1052. 26270260 10.1021/acs.chemrev.5b00188

[24] H. Langhals , O. Krotz , K. Polborn , P. Mayer , Angew. Chem. Int. Ed. 2005, 44, 2427–2428; 10.1002/anie.200461610 15770634

[25] B. Zhang , H. Soleimaninejad , D. J. Jones , J. M. White , K. P. Ghiggino , T. A. Smith , W. W. H. Wong , Chem. Mater. 2017, 29, 8395–8403.

[26] S. Nakazono , Y. Imazaki , H. Yoo , J. Yang , T. Sasamori , N. Tokitoh , T. Cédric , H. Kageyama , D. Kim , H. Shinokubo , A. Osuka , Chem. Eur. J. 2009, 15, 7530–7533. 19575351 10.1002/chem.200901318

[27] M.-J. Lin , A. J. Jimenez , C. Burschka , F. Würthner , Chem. Commun. 2012, 48, 12050–12052. 10.1039/c2cc36719j 23104274

[28] A. J. Jiménez , M.-J. Lin , C. Burschka , J. Becker , V. Settels , B. Engels , F. Würthner , Chem. Sci. 2014, 5, 608–619.

[29] J. Gierschner , Phys. Chem. Chem. Phys. 2012, 14, 13146–13153. 22941317 10.1039/c2cp42057k

[30] E. Sebastian , A. M. Philip , A. Benny , M. Hariharan , Angew. Chem. Int. Ed. 2018, 57, 15696–15701; 10.1002/anie.201810209 30338635

[31] F. Cacialli , J. S. Wilson , J. J. Michels , C. Daniel , C. Silva , R. H. Friend , N. Severin , P. Samori , J. P. Rabe , M. J. O'Connell , P. N. Taylor , H. L. Anderson , Nat. Mater. 2002, 1, 160–164. 12618803 10.1038/nmat750

[32] A. Rose , Z. Zhu , C. F. Madigan , T. M. Swager , V. Bulovic , Nature 2005, 434, 876–879. 15829959 10.1038/nature03438

[33] D. M. Eisele , J. Knoester , S. Kirstein , J. P. Rabe , D. A. Vanden Bout , Nat. Nanotechnol. 2009, 4, 658–663. 19809457 10.1038/nnano.2009.227

[34] D. Sahoo , K. Sugiyasu , Y. Tian , M. Takeuchi , I. G. Scheblykin , Chem. Mater. 2014, 26, 4867–4875.

[35] A. Leventis , J. Royakkers , A. G. Rapidis , N. Goodeal , M. K. Corpinot , J. M. Frost , D.-K. Bučar , M. O. Blunt , F. Cacialli , H. Bronstein , J. Am. Chem. Soc. 2018, 140, 1622–1626. 29337534 10.1021/jacs.7b13447

[36] A. A. Pakhomov , V. I. Martynov , Chem. Biol. Rev. 2008, 15, 755–764. 10.1016/j.chembiol.2008.07.009 18721746

[37] R. J. Cogdell , A. Gall , J. Köhler , Q. Rev. Biophys. 2006, 39, 227–324. 17038210 10.1017/S0033583506004434

[38] P. Kubelka , F. Munk , Z. Techn. Phys. 1931, 12, 593–601.

[39] F. C. Spano , Acc. Chem. Res. 2010, 43, 429–439. 20014774 10.1021/ar900233v

[40] T.-S. Ahn , R. O. Al-Kaysi , A. M. Müller , K. M. Wentz , C. J. Bardeen , Rev. Sci. Instrum. 2007, 78, 086105. 17764365 10.1063/1.2768926

[41] A. Liess , M. Stolte , T. He , F. Würthner , Mater. Horiz. 2016, 3, 72–77.

[42] J. L. West , G. R. Magyar , J. R. Kelly , S. Kobayashi , Y. Iimura , N. Yoshida , Appl. Phys. Lett. 1995, 67, 155–157.

[43] S. W. Culligan , . Y. Geng , S. H. Chen , K. Klubek , K. M. Vaeth , C. W. Tang , Adv. Mater. 2003, 15, 1176–1180.

[44] T. Nishizawa , H. K. Lim , K. Tajima , K. Hashimoto , J. Am. Chem. Soc. 2009, 131, 2464–2465. 19178280 10.1021/ja810123b

[45] B. C. Campbell , G. A. Petsko , C. F. Liu , Structure 2018, 26, 225–237. 29307487 10.1016/j.str.2017.12.006

[46] J. H. Park , P. Scheerer , K. P. Hofmann , H.-W. Choe , O. P. Ernst , Nature 2008, 454, 183–187. 18563085 10.1038/nature07063

[47] V. Gradinaru , N. C. Flytzanis , Nature 2016, 529, 469–470. 26819038 10.1038/529469a

[48] T. Brixner , J. Stenger , H. M. Vaswani , M. Cho , R. E. Blankenship , G. R. Fleming , Nature 2005, 434, 625–628. 15800619 10.1038/nature03429

[49] J. Wen , H. Zhang , M. L. Gross , R E. Blankenship , Biochemistry 2011, 50, 3502–3511. 21449539 10.1021/bi200239k PMC4000732

[50] CCDC 1901807 contains the supplementary crystallographic data for this paper. These data can be obtained free of charge from The Cambridge Crystallographic Data Centre.

