# Supplementary material for: Protein‐like Enwrapped Perylene Bisimide Chromophore as a Bright Microcrystalline Emitter Material
Source: Angew Chem Int Ed Engl. 2019 Aug 12;58(38):13385–9. doi: 10.1002/anie.201907618 (PMC6772080; doi:10.1002/anie.201907618)
Supplement: Supplementary file 1 — Supplementary [file ANIE-58-13385-s001.pdf]

Angewandte Chemie
Eine Zeitschrift der Gesellschaft Deutscher Chemiker

Supporting Information

Protein-like Enwrapped Perylene Bisimide Chromophore as a Bright Microcrystalline Emitter Material

*David Schmidt[+], Matthias Stolte[+], Jasmin Süß, Andreas Liess, Vladimir Stepanenko, and Frank Würthner**

anie_201907618_sm_miscellaneous_information.pdf

Table of Contents

Experimental Procedures 3
Results and Discussion 5
Supporting References 12
Author Contributions 12

Experimental Procedures

Synthesis. All solvents and reagents were obtained from commercial sources and used without further purification unless stated otherwise. Column chromatography was performed on silica gel (particle size 0.040−0.063 mm) with freshly distilled solvents as eluents. ^{1}H and ^{13}C NMR spectra were recorded on a Bruker Avance III HD 400 MHz spectrometer. ^{13}C{^{1}H} NMR spectra are broad band proton decoupled. All chemical shifts (δ) are given in parts per million (ppm) and are reported relative to tetramethylsilane (TMS). Coupling constants (J) are quoted in Hertz (Hz). Spectra are referenced internally to residual proton solvent resonances or natural abundance carbon resonances. MALDI-ToF mass spectra were recorded on a Bruker Daltronik GmbH (Autoflex II) mass spectrometer. Trans-2-[3-(4-tert-butylphenyl)-2-methyl-2-propenylidene]-malononitrile (DCTB) was used as matrix. High-resolution ESI-ToF mass spectrometry measurements were carried out on a microToF focus instrument (Bruker Daltronik GmbH). Elemental analysis was performed with an Elementar vario micro cube.

N,*N*‘-Dicyclohexyl-1,7-dibromoperylene-3,4:9,10-tetracarboxylic acid bisimide was prepared according to a literature procedure[S1].

2,4,6-Tris(4-*tert*-butylphenyl)phenol was prepared according to a modified literature procedure (Scheme S1)[S2].

2,4,6-Tribromophenol (1.29 g, 3.90 mmol), 4-*tert*-butylphenylboronic acid (2.46 g, 13.8 mmol), tris(dibenzylideneacetone)dipalladium(0) (114 mg, 124 μmol) and triphenylphosphine (291 mg, 1.11 mmol) were suspended in a degassed mixture of 120 ml toluene, 21 ml methanol and 21 ml of an aqueous 2 M sodium bicarbonate solution under an atmosphere of nitrogen. Subsequently, the reaction mixture was heated to 75 °C for 19 h and after being cooled down to room temperature the organic layer was separated. The aqueous solution was extracted twice with each 100 ml dichloromethane and the combined organic layers were dried over sodium sulfate. After evaporation of the solvent under reduced pressure, the crude product was suspended in methanol and collected by filtration to yield 2,4,6-tris(4-*tert*-butylphenyl)phenol as a colorless solid.

Yield: 72 % (1.37 g, 2.79 mmol). The analytical data correspond to those reported in literature[S2].

***N,N*‘ - Dicyclohexyl - 1,7 - (2,4,6 - tris(4 - *tert* - butylphenyl)phenoxy)perylene - 3,4:9,10- tetracarboxylic acid bisimide (PBI 1**; Scheme S1).

A suspension of *N*,*N*‘-dicyclohexyl-1,7-dibromoperylene-3,4:9,10-tetracarboxylic acid bisimide (175 mg, 245 μmol), 2,4,6-tris(4-*tert*-butylphenyl)phenol (1.20 g, 2.45 mmol) and cesium carbonate (798 mg, 2.45 mmol) in 35 ml degassed NMP was heated to 80 °C for 2 h under an atmosphere of nitrogen. After being cooled down to room temperature, 200 ml 2 M hydrochloric acid were added to the reaction mixture and the solid was collected by filtration, washed three times with each 30 ml water, three times with each 20 ml methanol and subsequently dried under vacuum. The crude product was purified by silica gel column chromatography (eluent: dichlormethane:hexane 4:6, 2,4,6-tris(4-*tert*-butylphenyl)phenol eluted as the first colorless band and **PBI 1** as the second orange band) and precipitated from a concentrated dichloromethane solution by addition of hexane to yield **PBI 1** as an orange solid. Single crystals of **1** could be grown by slow evaporation of dichloromethane from a solution of **PBI 1** in dichloromethane:hexane 3:7 at room temperature.

Yield: 78 % (298 mg, 195 μmol). **EA**: $C_{108}H_{110}N_2O_6$ (1532.08): found (calculated) C 84.15 (84.67), H 7.39 (7.24), N 1.86 (1.83). **MP**: >350 °C **MS** (MALDI-ToF, neg. mode, DCTB, dichloromethane): m/z: calculated for $C_{108}H_{110}N_2O_6$: 1530.836 [M]$^{-}$, found: 1530.540. **HRMS** (ESI-ToF, pos. mode, acetonitrile/chloroform 1:1): m/z: calculated for $C_{108}H_{111}N_2O_6$: 1531.84421 [M+H]$^{+}$, found: 1531.84245. **UV/vis** (CH_2Cl_2): λ_{max}/nm (ε_{max}/l mol^{-1} cm^{-1}) = 559 (72000), 519 (40350), 485 (13850). **Fluorescence** (CH_2Cl_2): λ_{max}/nm = 580; $\Phi_{Fl.}$ = 100 %. **CV** (CH_2Cl_2, 0.1 M NBu_4PF_6, vs. Fc^+/Fc): $E_{1/2}^{red}(X^-/X^{2-})$ = –1.73 V, $E_{1/2}^{red}(X/X^-)$ = –1.44 V, $E_{1/2}^{ox}(X/X^+)$ = 0.80 V. **^{1}H NMR** (400.1 MHz, CD_2Cl_2, 300 K): δ/ppm = 0.93 (s, 36 H, C*H*$_3$), 1.30 (m, 3 H, C*H*$_2$), 1.38 (s, 18 H, C*H*$_3$), 1.44 (m, 3 H, C*H*$_2$), 1.72 (m, 6 H, C*H*$_2$), 1.89 (m, 4 H, C*H*$_2$), 2.50 (m, 4 H, C*H*$_2$), 4.94 (m, 2 H, NC*H*), 6.96 (m, 8 H, aryl-C*H*), 7.35 (m, 8 H, aryl-C*H*), 7.53 (m, 4 H, aryl-C*H*), 7.68 (m, 4 H, aryl-C*H*), 7.73 (s, 4 H, aryl-C*H*), 7.92 (s, 2 H, aryl-C*H*), 8.43 (d, 2 H, $^{3}J_{HH}$ = 8.4 Hz, aryl-C*H*), 9.39 (d, 2 H, $^{3}J_{HH}$ = 8.4 Hz, aryl-C*H*). **^{13}C NMR** (100.6 MHz, CD_2Cl_2, 300 K): δ/ppm = 26.9 (*C*H$_2$), 29.5 (*C*H$_2$), 31.1 (*C*H$_3$), 31.5 (*C*H$_3$), 34.5 (*C*CH$_3$), 34.9 (*C*CH$_3$), 54.0 (N*C*H), 121.4 (aryl-*C*H), 121.9 (aryl-*C*), 122.3 (aryl-*C*), 123.6 (aryl-*C*), 124.3 (aryl-*C*), 125.1 (aryl-*C*H), 126.3 (aryl-*C*H), 127.1 (aryl-*C*H), 128.6 (aryl-*C*H), 129.1 (aryl-*C*H), 129.3 (aryl-*C*H), 129.5 (aryl-*C*H), 133.7 (aryl-*C*), 134.9 (aryl-*C*), 136.4 (aryl-*C*), 137.3 (aryl-*C*), 139.2 (aryl-*C*), 148.5 (aryl-*C*), 150.8 (aryl-*C*), 151.2 (aryl-*C*), 155.9 (aryl-*C*), 163.6 (*C*O), 164.2 (*C*O).

Microcrystal growth: Micrometer-sized crystals of **PBI 1** were grown on a carbon covered copper grids (SAED, XRD), silicon wafer Si/SiO_2(100 nm) (XRD, OPM, AFM) or on quartz substrates (OPM) from dichloromethane:hexane 4:6 solution.

UV/vis spectroscopy: UV/vis spectra in CH_2Cl_2 solution (10^{-5} M, spectroscopic grade, Uvasol®, Merck) were measured at 298 K in a 10 mm cuvette (SUPRASIL®, Hellma® Analytics) using a Lambda 950 UV/vis/NIR spectrometer from Perkin Elmer equipped with a Peltier system P1+1. Absorption profiles of microcrystals in a $BaSO_4$ trituration were obtained by measuring the reflection spectra with a Perkin Elmer Lambda 950 UV/vis/NIR spectrometer equipped with an integration sphere. The reflection data were transformed according to Kubelka and Munk[S3] to obtain the absorption profile of the ensemble of the microcrystals.

Fluorescence spectroscopy: Fluorescence spectra in CH_2Cl_2 solution (10^{-7} M, spectroscopic grade, Uvasol®, Merck) were measured at room temperature on an Edinburgh Instruments FLS980-D2D2-ST spectrometer under magic angle conditions (54.7°) and were

corrected against the photomultiplier sensitivity and the lamp intensity. Localized fluorescence spectra of individual single crystals were measured on a Zeiss Axio Imager optical polarization microscope equipped with a Zeiss CCD spectrometer with a customized spot resolution of up to 1 x 1 µm^2 (A. S. & Co GmbH). The sample temperature was precisely controlled using a Linkam LTS420E heating stage and a Linkam T95-LTS system controller. Absolute fluorescence quantum yields of **PBI 1** in CH_2Cl_2 solution or as crystalline powder were determined on a Hamamatsu Absolute PL Quantum Yield Measurement System CC9920-02. The system is composed of a 150 W CW Xenon lamp as excitation source, a monochromator (250-700 nm, *FWHM* 10 nm), an integrating sphere, and a multi-channel spectrometer capable of simultaneously measuring multiple wavelengths between 300 and 950 nm. The determined apparent absolute quantum yields (Φ_{app}) were corrected for reabsorption and reemission[S4] using reabsorption-free fluorescence spectra recorded either with an Edinburgh Instruments FLS980-D2D2-ST spectrometer (CH_2Cl_2 solution) or with a Zeiss Axio Imager optical polarization microscope equipped with a Zeiss CCD spectrometer (microcrystals). Fluorescence lifetimes were determined with an EPL picosecond pulsed diode laser (λ_{ex} = 505 nm) for time correlated single photon counting (TCSPC) with an Edinburgh Instruments FLS980-D2D2-ST spectrometer under magic angle conditions (54.7°). Crystalline powders were aligned and investigated with a front face sample holder F-J03.

Cyclic voltammetry: The CV measurements were performed with a standard electrochemical analyzer (EC epsilon, BAS instruments, UK) in a three electrode single-compartment cell (glassy carbon electrode). The measurements were carried out under argon in a 2 x 10^{-4} M CH_2Cl_2 (HPLC grade, J.T. Baker, dried over calcium hydride and degassed) solution with ferrocene as internal standard. As supporting electrolyte, tetrabutylammonium hexafluorophosphate (NBu_4PF_6) was used at a scanning rate of 100 mV s^{-1}.

Single crystal structure determination: Single crystal X-ray diffraction data for **PBI 1** were collected at 100 K on a Bruker D8 Quest Kappa diffractometer with a Photon100 CMOS detector and multi-layered mirror monochromated CuK_α radiation. The structure was solved using direct methods, expanded with Fourier techniques and refined with the *SHELX* software package[S5]. All non-hydrogen atoms were refined anisotropically. Hydrogen atoms were included in the structure factor calculation on geometrically idealized positions. Crystallographic data have been deposited with the Cambridge Crystallographic Data Centre as supplementary publication no. CCDC1901807 (PBI 1). These data can be obtained free of charge from The Cambridge Crystallographic Data Centre via www.ccdc.ac.uk/data.request/cif.

XRD: Powder X-ray diffraction data of the isolated bulk material of **PBI 1** were collected at room temperature on a Bruker D8 Discover diffractometer with a LynxEye-1D-detector using CuK_α radiation (unsplit $K_{\alpha1}$ + $K_{\alpha2}$ doublet, mean wavelength λ = 1.5419 Å). Measurements were done in reflection geometry in coupled two theta/theta mode with a step size of 0.025° in 2θ and 0.25 s measurement time per step in the range of 5–50° (2θ) The experimental diffractogram was compared with a diffractogram simulated from single crystal X-ray data using the program Mercury[S6].

AFM: Atomic force microscopy images were obtained in the tapping mode with a NT-MDT SOLVER NEXT instrument equipped with silicon cantilevers (OMCL-AC160TS, Olympus) with a spring constant of 42 N m^{-1}.

SAED: Transmission electron microscopy and selected area electron diffraction (SAED) measurements were performed using a FEI Titan 80–300 transmission electron microscope at an accelerating voltage of 300 kV. Software JEMS V.4 was used for the simulation of the electron deflection pattern.

Results and Discussion

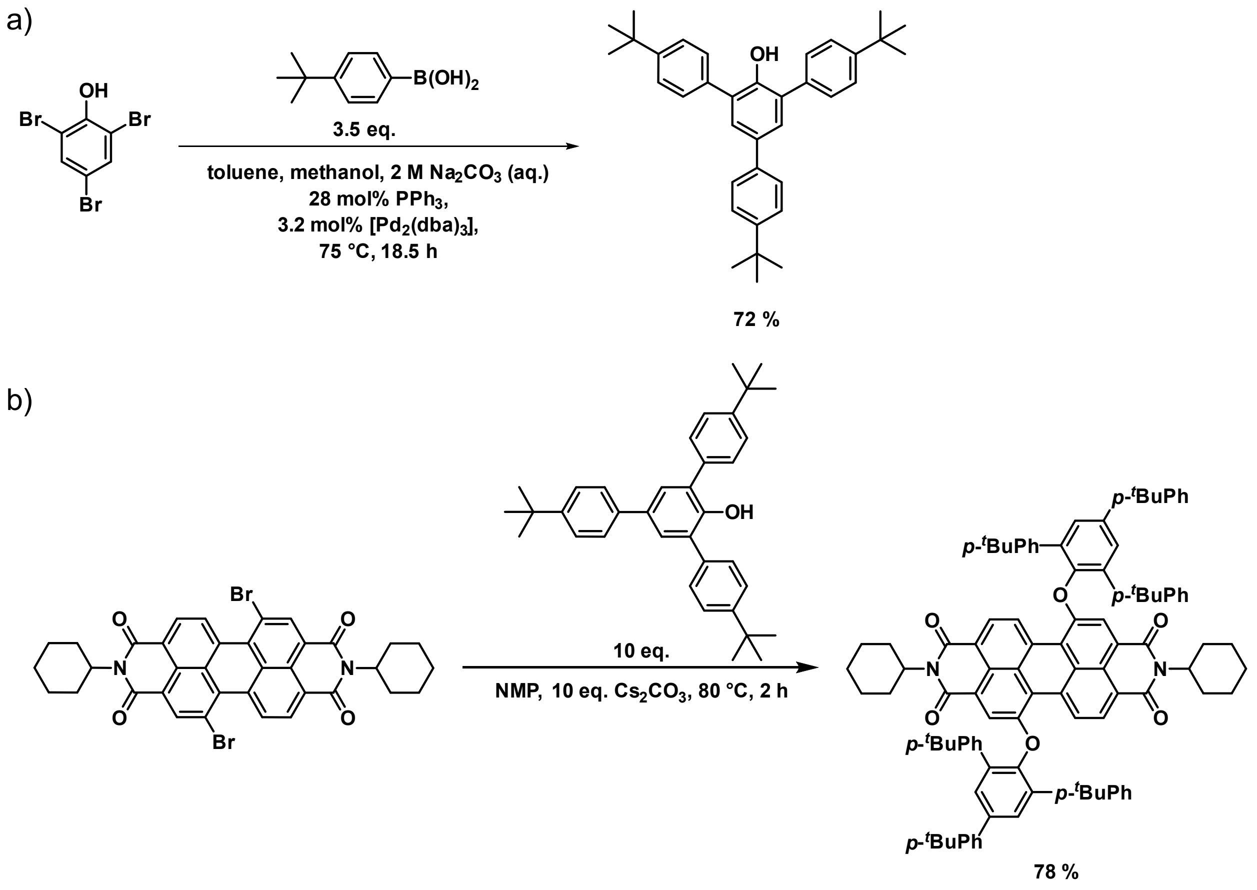

Scheme S1. Syntheses of a) 2,4,6-tris(4-tert-butylphenyl)phenol and b) **PBI 1**.

a)

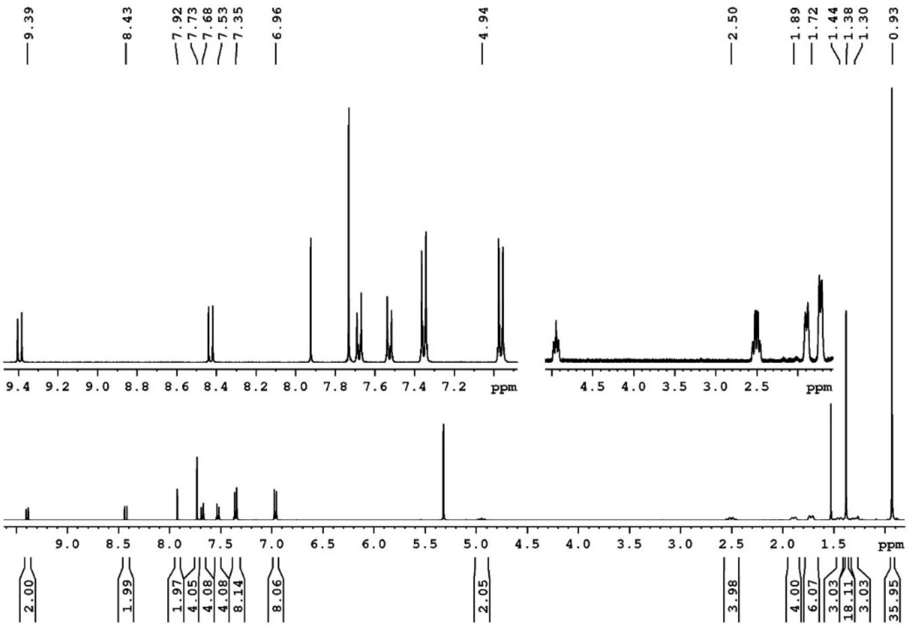

b)

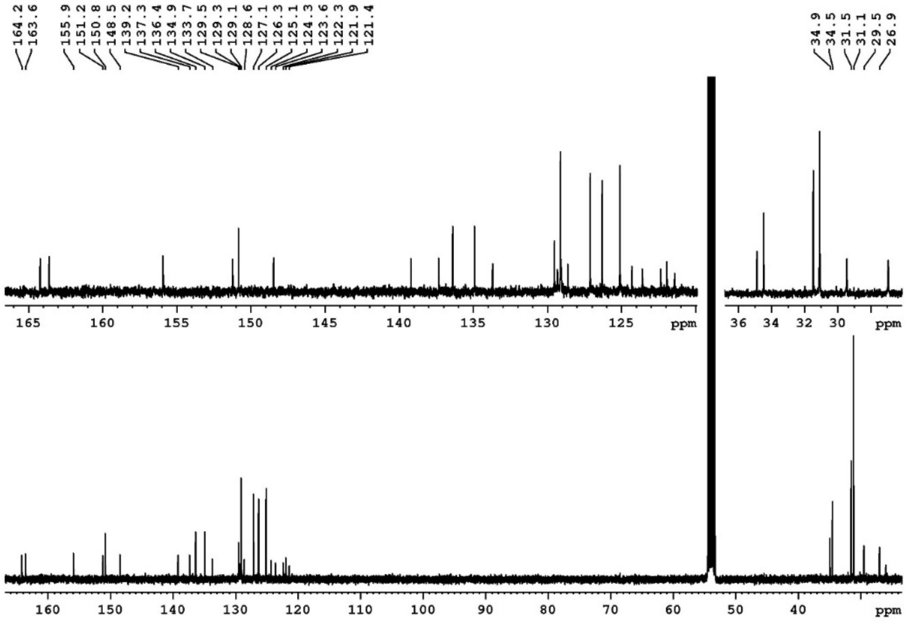

Figure S1. a) ^{1}H NMR spectrum of **PBI 1** at 300 K (400.1 MHz, CD_2Cl_2) and b) ^{13}C NMR spectrum of **PBI 1** at 300 K (100.6 MHz, CD_2Cl_2).

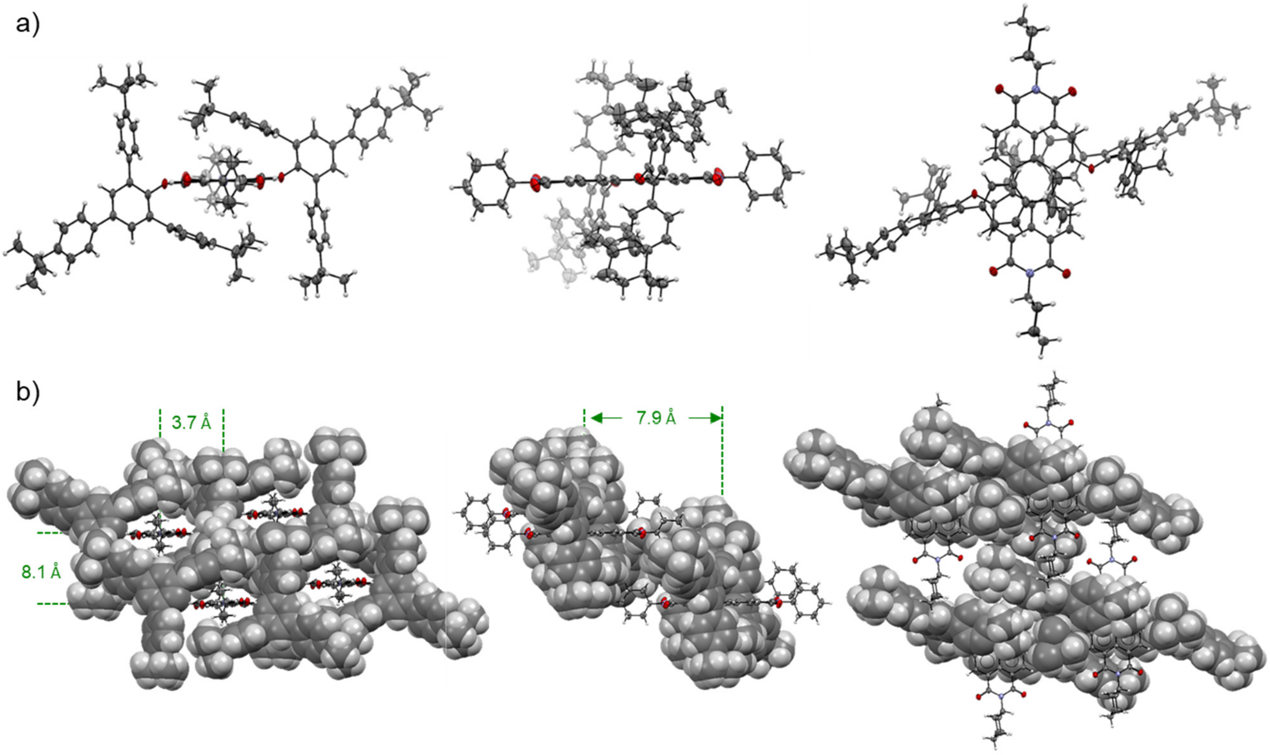

Figure S2. a) Structure of **PBI 1** determined by single crystal X-ray diffraction and b) arrangement of **PBI 1** in the solid state (left: view along the long molecular axis, middle: view along the short molecular axis, right: top view; ellipsoids set at 50% probability for the PBI π-scaffold, grey = carbon, white = hydrogen, blue = nitrogen, red = oxygen; 2,4,6-tris(4-*tert*-butylphenyl)phenoxy substituents are illustrated as space filling model in (b).

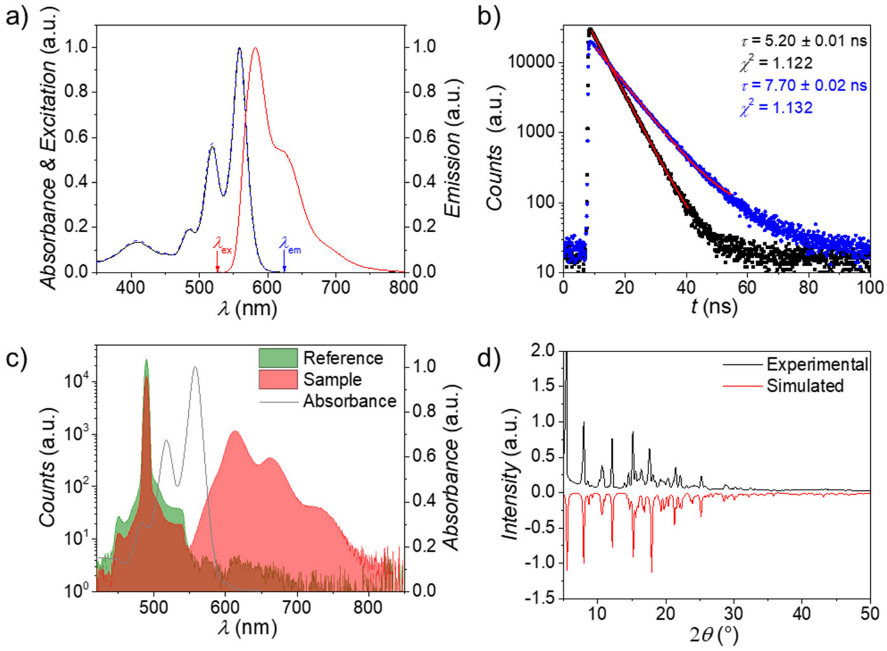

Figure S3. a) Normalized absorption (black solid line), excitation (blue dashed line, λ_{em} = 625 nm) and fluorescence (red solid line, λ_{ex} = 525 nm) spectra of **PBI 1** in a highly diluted dichloromethane solution (c_0 = 10^{-7} M) at 298 K. b) Fluorescence decay curves of **PBI 1** in a highly diluted dichloromethane solution (c_0 = 10^{-7} M, black squares) and as crystalline powder (blue circles, front face setup) at 298 K measured under magic angle conditions (54.7°) after excitation with a EPL laser diode at 505 nm. c) Optical profiles of an integrating sphere measurement of reference (green shaded area) and sample of **PBI 1** as crystalline solid-state material (red shaded area) at 298 K upon excitation at λ_{ex} = 490 nm. The normalized absorption profile of an ensemble of microcrystals (grey solid line) is displayed as well. d) Powder X-ray diffractogram of the bulk material of **PBI 1** (black line) studied in (c) and its simulated diffractogram (red line) by using the program Mercury obtained from single crystal X-ray data.

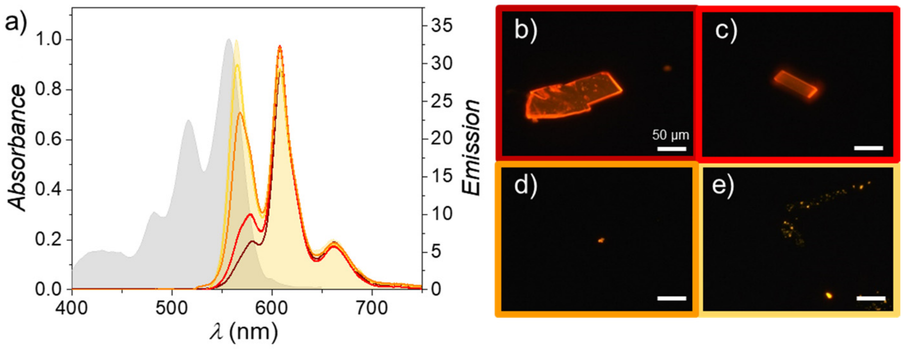

Figure S4. a) Emission spectra (solid lines) and fluorescence microscope pictures (b-e) of **PBI 1** single crystals of different sizes and thicknesses, marked with the same color code. The grey and light yellow areas in (a) mark the absorption and reabsorption-free emission profiles, respectively.

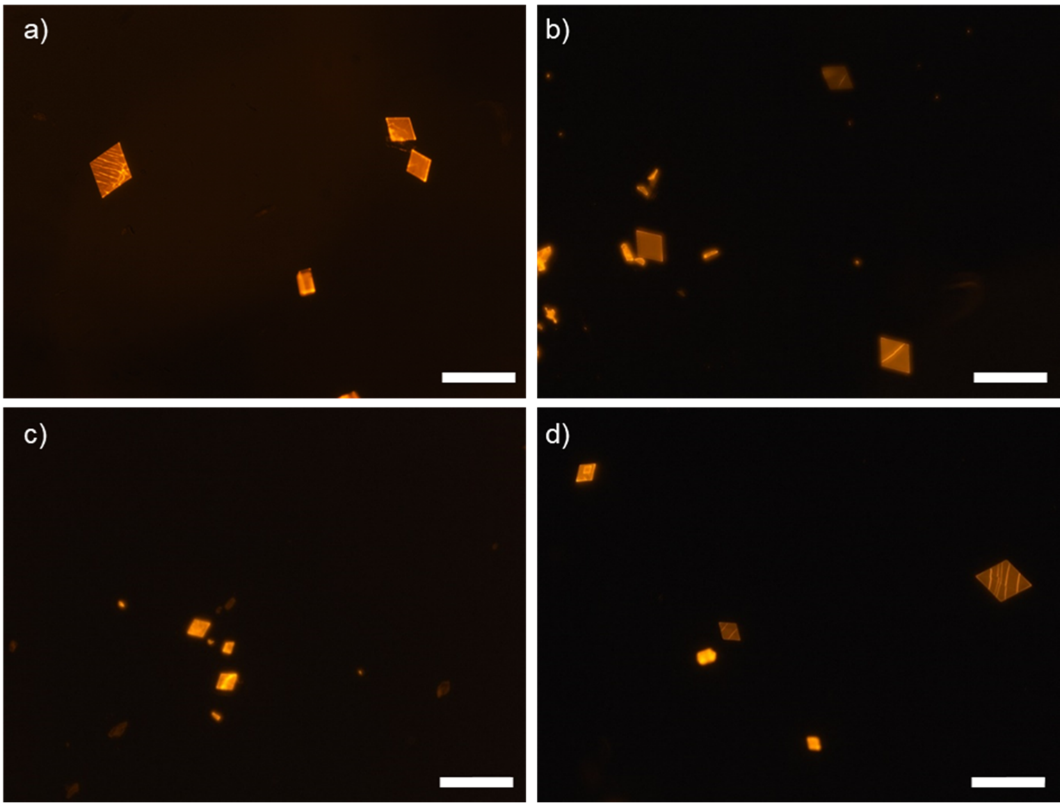

Figure S5. a)-d) Fluorescence microscope pictures of rhomboid microcrystals of **PBI 1** on quartz substrates under illumination using a filter cube (excitation: 530-580 nm, detection: > 600 nm) without polarizer and analyzer. Scale bar: 100 μm.

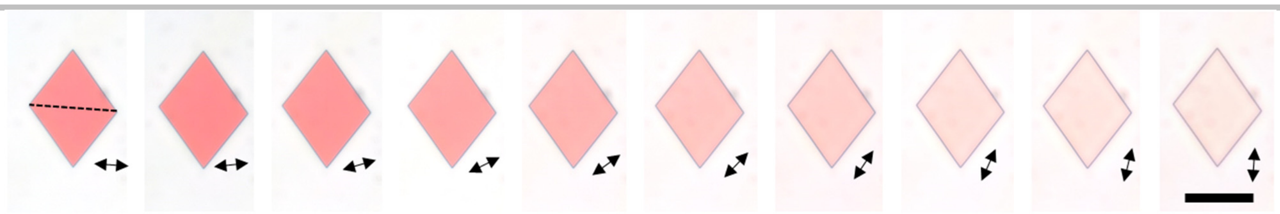

Figure S6. Polarization-dependent bright-field optical microscope pictures of a **PBI 1** microcrystal on a quartz substrate while rotating the polarization axis of the transmitted light in steps of 10° as indicated by the arrow. The dashed line indicates the orientation of the long molecular axis of the PBIs which is parallel to the transition dipole moment of the $S_0 \rightarrow S_1$ transition. Scale bar: 50 µm.

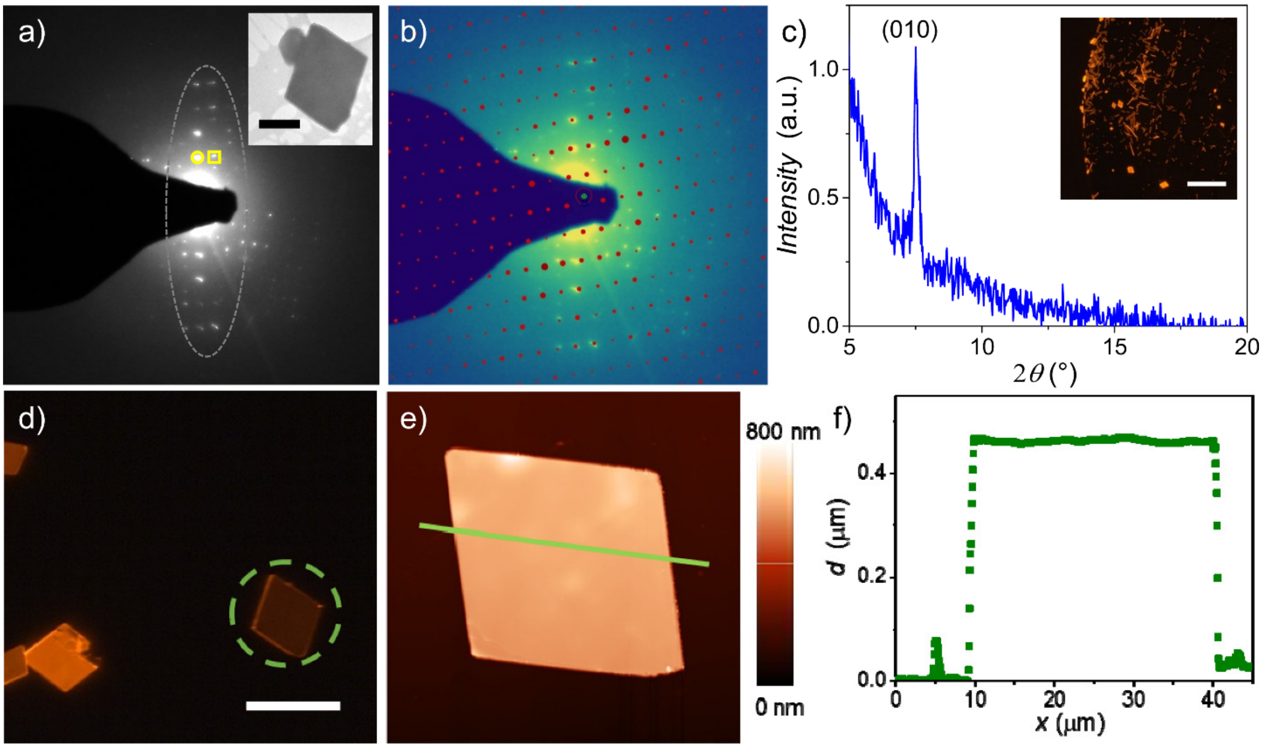

Figure S7. a) SAED patterns of a **PBI 1** microcrystal with marked reflexes (circle: (100), square: (001)). Inset: TEM image of the respective microcrystal on a Lacey carbon film; scale bar: 1.5 µm. b) Experimental SAED patterns (yellow-blue background) superimposed by the simulated electron deflection pattern (red dots) for the (010) plane parallel to the substrate surface. c) XRD of an ensemble of **PBI 1** microcrystals on a Lacey carbon film showing the (010) reflex parallel to substrate surface (Inset: Fluorescence microscopy picture of the respective sample, scale bar: 200 µm). d) Fluorescence microscopy image of a rhomboid microcrystal of **PBI 1** on quartz substrate. e) AFM image of the respective microcrystal in d (50 x 50 µm^2). F) Cross section analysis by AFM revealed a thickness of about 456 ± 11 nm.

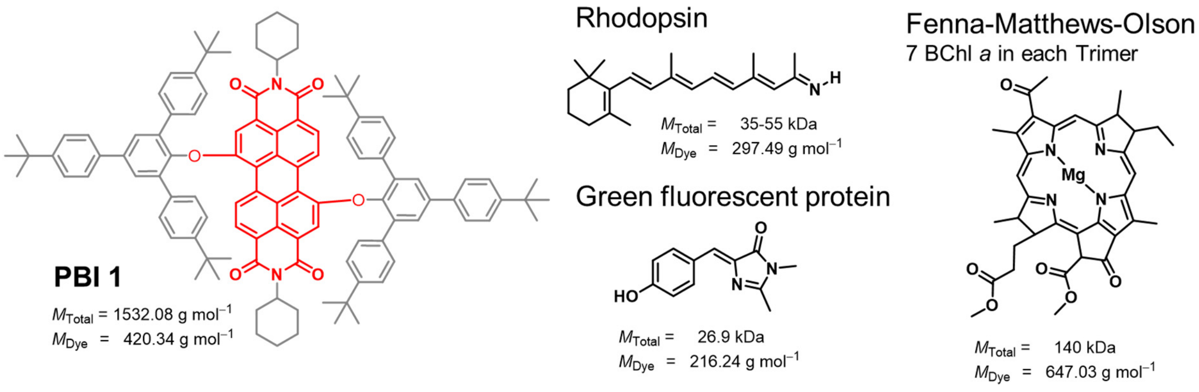

Figure S8. Comparison of the relative molecular mass content of chromophore (M_{Dye}) in respect to the total mass (M_{Total}) for **PBI 1** as well as protein-embedded rhodopsin, green fluorescent protein and Fenna-Matthews-Olson complex.

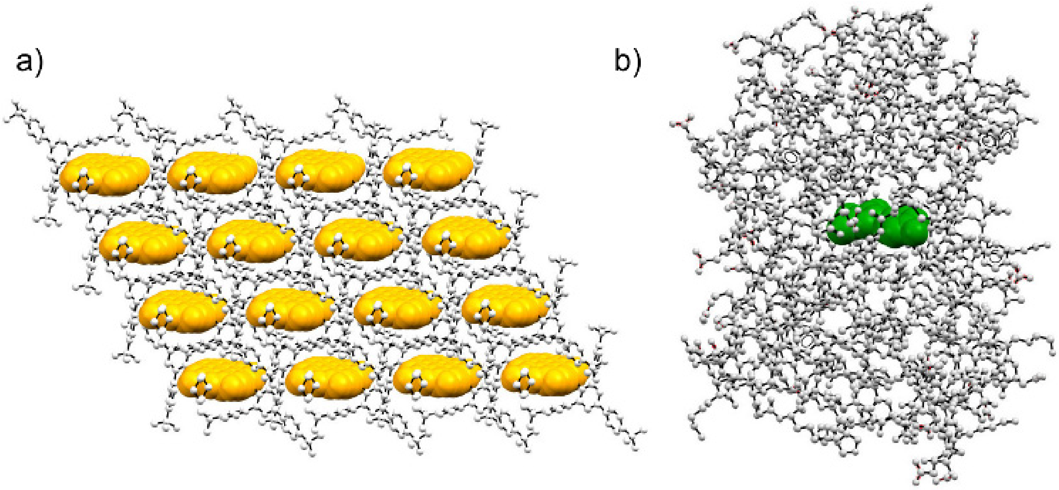

Figure S9. a) Packing arrangement of an array of **PBI 1** (24.5 kDa) in the single crystal structure in contrast to b) green fluorescent protein Clover mutant S147C/Q204C (**GFP**; 26.9 kDa). Chromophores are displayed as space filling model and highlighted in orange and green, respectively, while the 2,4,6-tris(4-*tert*-butylphenyl)phenoxy substituents of **PBI 1** and the polymer backbone of the **GFP** is colored in grey.

Table S1. Crystallographic data of **PBI 1**.

**Compound**	**PBI 1**
CCDC number	1901807
Chemical formula	$C_{108}H_{110}N_2O_6$
M / g mol^{-1}	1531.97
Crystal description	block
Crystal color	orange
Crystal system	triclinic
Space group	$P\bar{1}$
a / Å	11.8686(11)
b / Å	12.0737(9)
c / Å	16.5869(14)
α / °	98.614(4)
β / °	98.777(5)
γ / °	106.815(4)
V / Å^3	2200.9(3)
Z	1
ρ_{cal} / g cm^{-3}	1.156
μ / mm^{-1}	0.543
F(000)	820
Measurement range of *θ* / °	≤ 73.962
Completeness / %	98.3
Number of independent reflections	8654
Parametres/restraints	606 / 31
Goodness of fit for F^2	1.028
$R[I > 2\sigma(I)]$	R_1 = 0.0716<br>wR^2 = 0.1967
R(all data)	R_1 = 0.0874<br>wR^2 = 0.2113

Supporting References

[S1] F. Würthner, V. Stepanenko, Z. Chen, C. R. Saha-Möller, N. Kocher, D. Stalke, *J. Org. Chem.* **2004**, *69*, 7933-7939.

[S2] K. Dimroth, W. Tüncher, H. Kaletsch, *Chem. Ber.* **1978**, *111*, 264-271.

[S3] P. Kubelka, F. Munk, *Z. tech. Phys.* **1931**, *12*, 593-601.

[S4] T.-S. Ahn, R. O. Al-Kaysi, A. M. Müller, K. M. Wentz, C. J. Bardeen, *Rev. Sci. Instrum.* **2007**, *78*, 086105.

[S5] G. Sheldrick, *Acta Cryst. A* **2008**, *64*, 112-122.

[S6] C. F. Macrae, P. R. Edgington, P. McCabe, E. Pidcock, G. P. Shields, R. Taylor, M. Towler, J. van de Streek, *J. Appl. Crystallogr.* **2006**, *39*, 453-457.

Author Contributions

D.S. and J.S. conducted the synthesis and characterization (NMR, HRMS, EA, CV) of the **PBI 1**. D.S. grew the single crystal of **PBI 1** and performed all crystallographic studies. A.L. grew the oriented microcrystals on substrates and measured AFM. V.S. performed the SAED und TEM measurements. M.S. performed all spectroscopic studies and investigations by (fluorescence) optical polarization microscopy. F.W. conceived and supervised the whole work. All the authors contributed to the manuscript writing.
